# Adoption of digital twin for sustainable manufacturing and achievements of production strategic-planned goals

**DOI:** 10.1016/j.mex.2022.101920

**Published:** 2022-11-11

**Authors:** M.S. Jawad, Pavinraj Chandran, Azizul Azhar Bin Ramli, Hairulnizam Bin Mahdin, Zubaile Bin Abdullah, Mazidah Binti Mat Rejab

**Affiliations:** Fsktm Faculty, Uthm university, Malaysia

**Keywords:** Digital Twin, Strategic Planning, Adoption, Middleware Architecture, Product-Life-Cycle

## Abstract

To achieve the maximum return-of-investment for the adoption of Digital-Twin in manufacturing, organizations should be totally aware about the challenges that limit the widely adoption as well as opportunities that may create real-added values to their businesses at operational and strategic management. In this context, determining the most influential factors for successful adoption must be clear even at the early stages of planning towards high effective digital-transformation journey for business's sustainability. The beneficial achievements and outcome towards such successful planning and adoption of the industrial digital-twin are significant in terms of optimized processes, reduced costs and downtown of the operations, flexibility in product design and processes’ adaptation to satisfy future markets demands The main purpose of this paper is to propose adoption modelling of digital-twin for optimized products and production processes. The methodology of the proposed modelling can be considered unique in the following aspects of:

• Determining the expected added-values of adopting digital-twin to the manufacturing business according to certain business's operational criticality, budget and size.
• Allowing processes’ optimization at three levels of plant (factory) physical layout, Machines’ operational fault tolerance and final products’ design and quality.
• Allowing strategic-planning achievement for sustainable Production-Product and future demands.

## Graphical abstract

**Figure:**
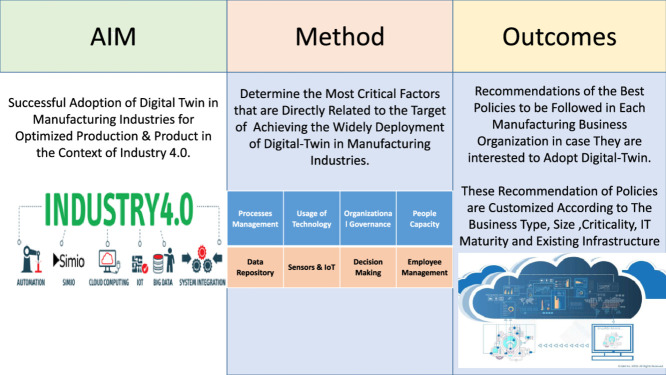

Specifications Table

**Table:** 

Subject area:	**Engineering**
More specific subject area:	*Industrial Digital Twin*
Name of your method:	*Digital-Twin Adoption Modelling for Optimized product-production*
Name and reference of original method:	*Name of the original modified method*: Metrics development and modelling the mixed reality and digital twin adoption in the context of Industry 4.0 ***Reference***: Sepasgozar, S. M., Ghobadi, M., Shirowzhan, S., Edwards, D. J., & Delzendeh, E. (2021). Metrics development and modelling the mixed reality and digital twin adoption in the context of Industry 4.0. *Engineering, Construction and Architectural Management*.
Resource availability:	https://www.emerald.com/insight/content/doi/10.1108/ECAM-10-2020-0880/full/html).

## Introduction

Digital twin can be defined as the digital replica of the physical assets and that can be also extended to include the processes and the movable objects as well as workers [1]. It can be considered as a great step towards the digital transformation in different domains of the construction management, hospitality. Healthcare, educational and manufacturing industries. The focus of this paper is on potentials of digital twin in smart manufacturing in the context of industry 4.0 and how to leverage these potential to the maximum levels. Integrating the 2D/3D digital replica with virtual reality, augmented reality and artificial intelligence extending the capability of digital-twin to very efficient human-machine interactions for the optimized performance as well as for real-time or near real-time decision-making and automation control. In brief, the full manipulation of the digital replica to reach to optimal performance is much more efficient and risk-free than the direct manipulation of the physical assets of the factories. In this context, digital-twins enable the best configuration and optimization of the assembly/production lines, also enable remote monitoring and interactive troubleshooting in real-time. Enabling virtual-commissioning to control and monitor certain machine operations and tolerance. Production and products can be dynamically adapted as a results of successful implementation of the digital-twin [2].

Cloud computing infrastructure is an important enabler technology of the overall digital-twin implementation picture. Simply, cloud computing can offer the data storage of the real-time IoT data collection for further data-analytics and construction of the digital-twin replicas.

## Digital twins alignment as strategic planning in industrial manufacturing enterprises

Businesses’ strategic planning involving visions, Goals and Initiatives for sustainability and market competitiveness and manufacturing enterprises are not exceptional from that [3]. To ensure sustainability of the manufactured product in medium-to-long terms, optimization of the resources, processes and response to the market dynamic demands as achievements of end-customers satisfactions are the heart of this strategic planning. In this era of industry 4.0 revolution, optimizing the performances at the levels of the factory layouts/processes as well as optimization of the productions and products are the key of success of the strategic planning of the manufacturing enterprises. Digitization for optimal automation controls and monitoring is considered to be recently the key performance indicator towards the manufacturing cost-saving, minimized waste, reduced down-time of the machines and flexible adaption for future production demands [4]. Digital-twin technology can be considered as the best solution towards this successful digital transformation journey in manufacturing processes and businesses. The next section discusses in details the different optimization level can be accomplished by applying digital-twin for smart manufacturing.

## The increased importance of digital-twin adoption in modern manufacturing

The successful adoption of the digital-twin can optimize manufacturing performance and processes at three different levels can be explained as the following. The first level optimization can be considered at the digital factory optimization. In this level, the optimization can be achievable in term the initial planning for the best layouts and architecture of the physical assets as well as the number of staffs. This optimization at the factory floor level can include as well the best shifts scheduling [5]. As a results of this level of optimization at the plant or factory floor level, a high return of capital and human investments can be feasible to aid the efforts of successful establishment of the businesses from the early stage even before the start of the production or tuning the existing operational performance to be optimal.

The next level of optimization that can be achieved by successful digital-twin adoption is considered at the production level [6,7]. Virtual commissioning is the most frequent term to describe this level of optimization. Virtual commissioning can be defined as involvement of virtual model that represents 3D simulation of the mechanical, electrical and control systems accurately and realistically. This 3D simulated model can be validated by the physical functions of a production system before the actual implementation. There are several considerable benefits of virtual commissioning as optimized production in terms of remote monitoring and maintenance also, reduced machine down-time and maximizing the operational tolerance.

The third-level of successful digital-twin adoption is at the product level. In this level, digital-twin aid the efforts of product adaptation for future demands in terms of volume and quality of the products [7,8]. Customers’ demands and markets dynamicity may require increased production and possible changes of products’ shape or materials. Minimizing or eliminating products defects can be considered also as the good contribution of digital-twin at this level.

As concluded remarks from the literature, digital-twin successful adoption can increase the production life-cycle in different phases’ aspects as the following:

- The design of the physical systems: In the concept of digital-twin product development life cycle, the designers have the ability to virtually explore design concepts and models then, enhance these concepts wherever lack of performance or shortfalls are discovered. Consolidating the physical and virtual worlds is added-value to the product's engineering as more flexibility to discover the optimal designs as well as the real-time data and updates of the different designs performance [9].
- In the system configurations / reconfigurations phase: digital-twin technology support and enable real-time testing and evaluation. Configurations can be synchronized and tuned to enable the validated system performance in a semi-physical simulation manner [10], [11].
- In the system operations, digital-twin technology optimizes the manufacturing processes in different aspects that can be explain as firstly; for maintenance processes, digital-twin allow real-time remote monitoring of the physical assets conditions and then alerts for schedule maintenance. This can lead to increased machines’ efficiency and life-time as well as reduced operational down-time. Another aspect of the operational efficiency that can be achieved is the virtual machine commissioning which can be achieved by digital-twin empowered augmented-reality capability for remote troubleshooting and machine-setup. This can reduce troubleshooting complexity and solving sudden machinery physical problems in faster ways with reduced man-power cost [12].
- In the level of products: digital-twin allows rapid prototyping for products’ new design and qualities requirements. This can add competitive advantage to satisfy end-client changing preferences and future market demands [13].

## Digital-twin as integrated added-values manufacturing in the context of industry 4.0 and industry 5.0

For increased efficiency, ease-of-use, automation capabilities with enhanced security aspects, digital-twin technology can be integrated with artificial-intelligence solutions, virtual/augmented reality. Recently blockchain technology can be utilized for distributed tokenized manufacturing processes in secure, trusted and enhanced traceability for collaborative and sustainable modern manufacturing.

Recently; the concepts of collaborative manufacturing and factory2factory communication are gaining increased intention for modern and smart manufacturing aspects in the context of industry 4.0. Blockchain empowered smart contract add the capabilities to tokenize and securely distribute the manufacturing experiences in consensus manner between manufacturing panels and nodes. The smart-contract technology adding to that the automated terms and conditions between contracted manufacturing parties so that the processes can be handled more accurately with minimum human-interventions [14].

Mass-individualization (also known as mass-personalization production) is another increasingly interested aspect of modern smart manufacturing technology. Some researchers recently believe that these aspects towards human-centric with sustainability and resilience are the path to the next industrial evolution namely industry 5.0. This coming industrial evolution is mainly aiming to deliver mass personalized products to customers with high speed and accuracy. Cloud-computing elasticity and highly tenability combined with blockchain and smart contract capabilities are the main enablers technology towards industry 5.0. This can be push forwards innovative mass individualized production and rapid prototyping services for circuit-boards production as an example [15,16].

## Digital twin middleware development as layered architecture

Although that recent digital twin technology is not yet middleware standardized architecture, it is still highly useful to understand and form some multi-layered architecture to give an idea about how the entire eco-system evolve around and interact during the implementation in this technology.

The first layer to be considered is the physical layer which define the devices’ connectivity as this layer define the different devices as sensors and the things connected to the IT as IoT devices. Also, in this layer the data aggregation as results of the collected data from these devices is determined with their associated gateways. In this layer as well, the digital twin be created from all these data aggregated from the different devices [8]. Devices’ connectivity is important issue to be defined in this layer as without these data the simulation which establish the digital twin cannot be accomplished as no real-time replica of the processes in equipment.

Next, a group of three layers as communication, information and functions layers are together defining the functions of data processing, data computing also, the analytics and management of the data. In the communication layer, the set of the communication protocols, the networks topologies, that network software to be used and the internet service providers are determined. Next to the communication layer is the information layer where actually the information will take place here as data modelling, event modeling, data storage and also the meta data. In top of the information layer in the functions layer where the actual functionalities taking place are defined in this layer. These functionalities are related to the streaming processes in cases where data is continuously generated from the IoT sensors. This requires to do event processing for the purposes of some important diagnostic issues such as to know the health conditions of the equipment. Usually these data are coming from remote places to be continuously analyzed so that events are happening have to be processed to generate an alerts. In some cases, the occurrence of multiple events may lead to the failures or performance degradation so such things should be mitigated or early resolved with the capabilities of the digital-twin. In short, as data are streaming and events coming in then multiple alerts can be analyzed immediately by the digital-twin to decide if there is a need for change in the environment settings to avoid risk of failures of performance degradation (bottlenecks in production lines). When events processing is carried-out then, they should be put in event processing policy place to determine what should be considered next as alert, reminder or failures to be treated. The visualization capability in the function layer allowing the capabilities to visualize and interact with the equipment in real-time through virtual reality (VR), augmented reality (AR) or the mixed reality (MR). In this layer as well, the dispatch from certain layer or orchestration of the layer functionalities are defined. The functionalities in this layer is extended as well by the capabilities of the analytics and machine learning algorithms for prediction purposes and to make sure that the system will perform well in the future [17].

After looking to the combination of the communication, information and function layers which focus on data processing, analytics and management, the next and the top layer is to be considered as the process layer. In the layer, the talk is focused on the business intelligence achievements as business connectivity, presentation and interaction. The focuses are on the governance, operation, management and the business applications. This is basically being the senior level and we are talking here about how the organization perform well or how the assembly lines of the production in the factory are performing well.

Adequate security has to be provided at the all levels. This is important issue to be considered always as all the machines and the entire system are maybe located in one center location or maybe spread among multiple factories and across different geographies so, here the data mining can be done in one location and the monitoring is done in another. For example, in hydraulic plant, mostly the Scada system which monitor the health of the connected machines although that the turbines are located in somewhere else as the flow of the water is happening in different location. As the data from all the different places have to be brought together, so network become important and hence security is also highly required here.

## Management services of the digital twin middleware architecture

This section explains the different services of the digital twin middleware. In this middleware there are various management components and in each of these component there are several possible services. These components are the virtual machine manager, interoperability, data management, model management and the services management [19]. The VM manager basically to help in carrying out front-simulation. It is used as a monitor service consists of things and event management services, it will carry-out simulation, decision-making and even control services, so the basic function can be considered as tracing, visualization, actuation and even conflict resolution or in case of duplicated data. Interoperability management component can be handled using semantic data interoperability services which includes service search and discovery. The data management can be handled by the help of data acquisition and knowledge discovery also called as the data management services which will be able to handle streaming data or batch processing including security [17]. Because data is streaming there is need to ensure to ensure that data is secure otherwise if the data is breached and they may be resulting in creating different error or wrong digital twin. The next component is the model management which focusing on data computation and representation services in terms of the behavior, semantic and behavioral modelling.

In term of services management, the major component which connect all components together is the connectivity service and these services include cyber threat analysis and detection also, ensuring that the data is only visible to the request group of users in the entire system.

## Considerations for successful digital twin adoption strategy

This section explains how a particular organization can behave in the best practices to adopt strategy of the digital twin technology. This strategy can be considered at three levels as kickstart, blueprint and then setting the real digital twin adoption long-term strategy.

### Kickstart as the first steps towards successful adoption

Here, the need for digital twin technology is highlighted by identifying the problem in the existing industry and how digital twin can contribute to solve or mitigate these problems. For example, the problem that can be highlighted as the poor quality or the lack of trust of products [21]. In this case, the digital twin can help in improving the products quality also can act as proof of authenticity and product integrity. Another example that can be considered is when there is sometime missing or tempered data about certain equipment in production lines that work at a production rate or when the case of production that should be in a particular quality. Here the digital twin can contribute to the solution by providing transparency and compliance throughout the production life-cycle. That can be understood as the digital twin can be tested out for the optimized plant layout, number of staffs and shifts in each assembly line as well as for best monitoring the equipment production tolerance and maintenance so, this technology is successfully delivering in the battle of production and complying with whatever quality and norms have been decided for the products. A good level of data transparency can be achieved as the data available for the digital twin is the same data where it is produced from the real environments.

Business intelligence in manufacturing enterprises can be highly achievable with the aids of the digital twin technology so, the organization can optimize the collected data to know what is the total stock and which parts or which types of the products selling more. This can be nicely aligned to the goals of objectives of the organization to adapt their products being delivered by changing the design or enhancing the quality, If the data is available from anywhere across the supply chain it will help organization to perform better and adapt to satisfy future demands. In brief, digital twin can enhance significantly the digital experiences of production and equipment as all the data is in the digital format so that the products and the assets will each have a unique digital story to help in giving authenticity, transparency, compliance and visibility [18].

The processes on how an organization kickstarting of the adoption of digital twin can be explained as the following: first; ideate, as process to discover opportunities where the digital twin can be used, second; identify which assets and equipment where digital twin will help in doing optimization at the earlier virtualized environment then select the appropriate processes for the equipment or the assets. Third; the development of the digital twin pilot which look at all the important aspects of the digitization so that the pilot is which makes the digital twin alive and it is a model-based system simulation that can define the different scenarios. Next step is to industrialize as finalized pilot with a closed-loop. It is important that after doing a pilot to get feedback and ensure that in the system, the pilot is the best digital twin functionalities. Scaling is important process to be considered so that the pilot can update the digital twin with all the required processes and features and ensure it is complete system solution. Final process is monitoring the processes of the digital twin different uses and it ensure whether it is working properly.

#### Implementation principles

Implementation principles of digital twin adoption strategy can be explained in the following points:

- Integrating IoT into existing or new ICT infrastructures.
- Connect the planned digital twin services to a centralized cloud-based location with big data streaming and analytics capabilities to collect the sensing data and enrich it with the business intelligence functionalities.
- Periodically data analysis to discover the improvements possibilities in products or business models.
- Intelligently use digital insights to create new innovative services.

Digital twin implementation is centralized around the three main concepts of involve of the entire product value chain, Include data from different sources and then establishment of well documentation practices [19].

### Digital twin blueprint

Digital twin blueprint is basically a design on paper and for each individual asset. This considers the main points should be thought of or precautions have to be taken and what characteristics have to be put for a digital blueprint of an individual asset [20].

Also, in this phase the failure modes should be clearly understood as a complete list of likely equipment failure modes and in which conditions. External and internal factors that have impact contributing to different type of failure should be identified as well. After failure modes of assets are identified, the set of strategies should be determined as optimized suggestions based on the class of assets.

Analytics is important part of the digital twin blueprint design as the predictive or the diagnostic analytics can enable early identification of failures. The includes health indicators that measures and manage the operational conditions of the devices and assets. Here metrics to identify asset KPIs are highly beneficial for understanding and characterizing the performance [21,22] . Life-cycle cost can be considered also as historical model and operational dataset using tools to estimate the asset life-cycle cost and performance expectations.

### Digital twin adoption strategy

The actual digital twins’ adoption strategy should be planned well as it is not standard to be followed across all organizations. It could be differ based on the type of the business as well as the size of the organization [23].

The first thing to be considered in the adoption strategy is a robust platform for different industrial application. Assets with same functionalities need to be configured in different ways depending on where they will be used. The configurations of these assets are decided based on the underlying and the optimum conditions. The solution here can be considered as separating of the digital twin so that there will be digital twins carefully created for each asset used in similar applications.

The method of the implementation approach should be considered carefully in the adoption strategy. The issue here is that, implementation of digital twin for the entire factory floor at once maybe is risky choice. The better alternative solution to be considered is to build digital twin for assets based on criticality and data dependency in a phased manner to achieve the target goal of a digital factory [24].

How to source quality data into the digital twin platform is a point of high concern here as the quality data matters. The answer of the question of how to tackle lack of common devices communication standards. This can be understood as IoT technologies have evolved over a period of time and they have come-out very fast but the common standard has not been yet fully defined. The best practices as adoption strategy solution to be followed can be highlighted as: following standard data collection templates, collecting good number of sampled data also using techniques to be sure of removing data duplications. Also, it is highly recommended to deal with services providers which adopt standard software framework which enables communication and interaction among IoT devices.

User education should be considered as well as the setup and the adoption of digital twin is developed based on factors such as skepticism and user resistance. This issue can be resolved by using properly documentation and provide training and software socialization which encourage digital twin adoption.

## Digital twin adoption questionnaire in manufacturing enterprises

Various categories should be considered in the design of the questionnaire to determine the levels of possible adoption of the digital-twin technology in manufacturing enterprises. The most important category to be considered from the beginning is the processes management. The first question might be asked here could be: Does your organization has data repository in term of CAD documents or any other forms of design documents? Are these documents manually or digitally stored? How the design documents are maintained? Is it like maintained in central local or in a distributed geographical location? Does the organization have central design repository? These types of questions are important as the digital-twin is going to use data and this data is coming from sources and has to be put into a repository. Quality data is always crucial to create digital-twin properly and when data is collected from different resources there is always possibilities for data duplication and then deduplication methods should be carried-out. Also, these data should be collected in one central-design repository then only then can be effectively used to build a digital twin [19].

In the term of processes management, it should be asked about how is the lifecycle management in an organization, is it documented? Is there are standardized engineering and manufacturing activities? Are they documented and explained to people? Are they updated in a regular basis? And how are the processed changes approved in the organization? Are these updates versioned? And how approve these changes? So for example if a junior engineer proposed changes on the processes in certain part of the cycle from his/her point of view, here in the interests of the organization as entire processes, it is highly possible that these processes changes have to be reviewed at multiple-levels before it can be approved.

Next in the term of processes management, we have to look to the business criticality. The questions to be asked here are: how the critical activities in operations handled? How they are documented and digitalized? Who is responsible, involved, reviewing and approving? criticality is important because an actions has to be taken, it can be checked in digital twin and then it can be implemented in the line and need to know exactly what to be done [20].

Another important aspect of the processes management is how to handle failure. Here need to determine how is strategy planning is carried-out? and how is mitigation issues are carried-out? What is processes, how they are handled and who is responsible for these processes? [25].

Processes management in term of the design. Whenever any particular entity product or process design is made, what are the processes to be followed? Is it 2D or 3D visualization? How these visualizations are maintained and how they are stored? What are the digital tools that are in use (such as CAD/CAM)? How these designs are stored and who can update them? [26].

Another main category to be considered in the design and formulation of the adoption questionnaire can be considered as the usage of technology. As in an organization what level of the technology is used? And how this technology is used? Like, what are the sensors used? How they are used and from which vendors these sensors been purchased? What are the quality of these sensors? How is the data collected and stored from these sensors? How is the security of the collected data is maintained? Also, how is the failures can be handled in terms of the sensors and IoT ?

Connectivity is another important aspect to be explored under the category of the technology usage like, how is the end-to-end data collection is accomplished and how is these data shared to the information systems? What is the main modes of communication for operation is being used, is it Wi-Fi, wired connection or Bluetooth? Also, the levels of integration with Management Information System (MISS), Decision Support System (DSS) and Enterprise Resource Planning System (ERP) with the engineering systems (as the engineering system is where the actual design will be happened) [27,28].

Organizational governance is an important category of the technology adoption to be considered and some of the questions can be asked here are: How is decision-making carried-out? Is it proactive or real-time decisions? Also, how is the periodic maintenance is carried-out? What about the data analytics processes that are used to derive the decisions? Strategic planning of an organization should be investigated as well with possible questions such as: is the strategic-planning in the organization is focusing in short-term or long-term or combinational of both? Is the planning mainly is focusing on the strengths or it based on competition? In brief, organizational governance is important to be clearly understood because if the points related to strategic-planning and decision-making in the organization are not taken into accounts, then it will be really not clear if the organization in need for digital-twin technology or not [29].

**Fig. 1:**
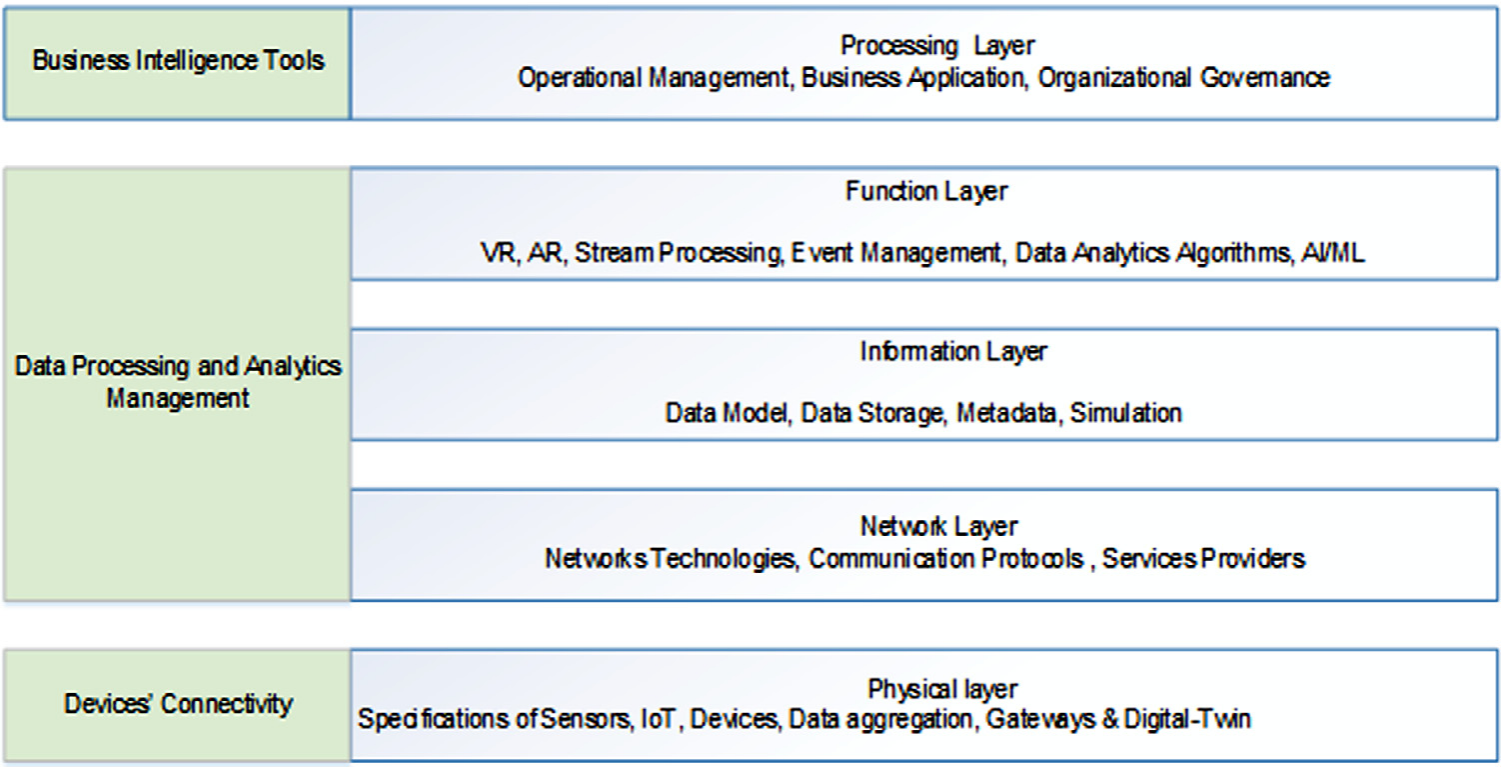
Digital Twin Business Intelligent Architecture.

**Fig. 2:**
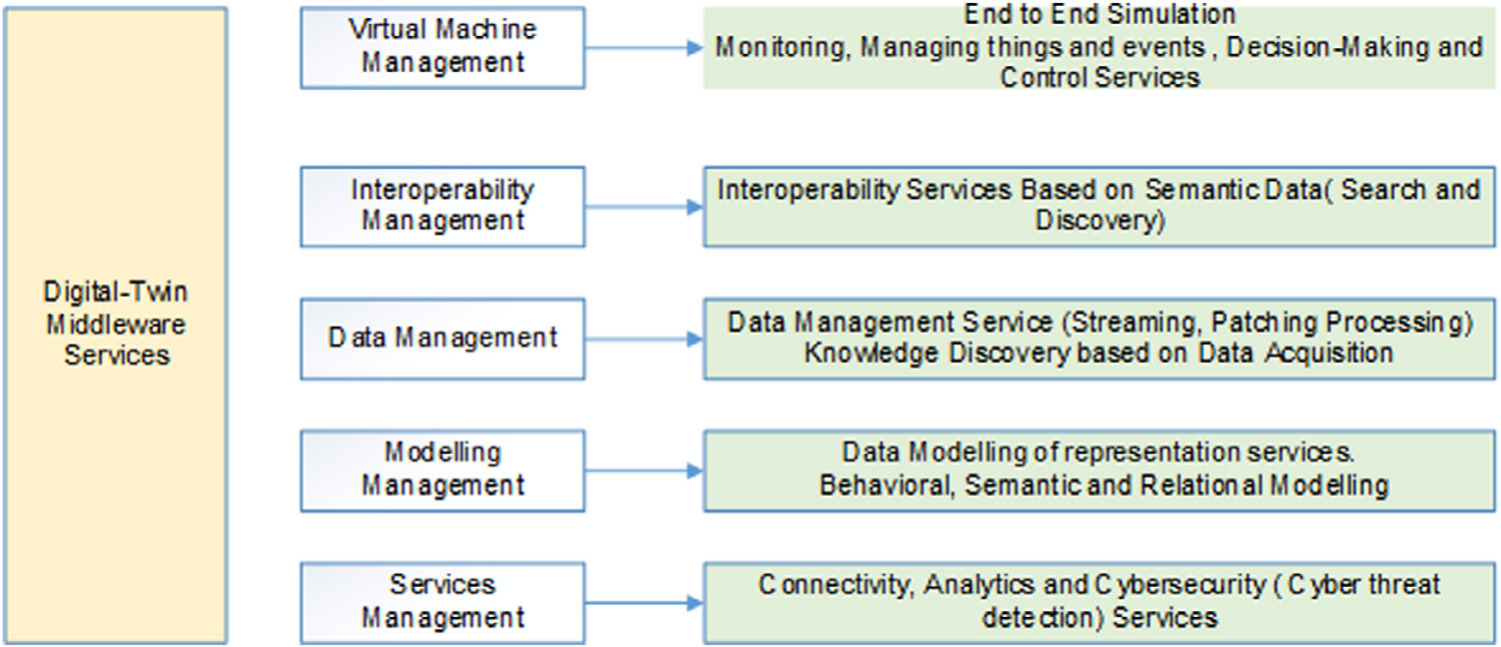
Digital Twin Middleware Layered Architecture.

There is also a set of question related to the people capability. Like, how's people management is carried-out? How's the employees training is carried-out? What are the policies and procedures for competency enhancements? And, what is the organizational level of the skills set as of now and how to leverage that to higher level and are the employee are capable to reach that? Table 1, Figs. 1, 2

**Table 1:** Influential factors in Digital-Twin Technology Adoption.

Processes Management	Usage of Technology	Organizational Governance	People Capacity
**Data Repository**	**Sensors & IoT**	**Decision Making**	**Employee Management**

• CAD Documents (Digital or Manual) • Design Documents (Centralized or Distributed) • Central Design Repository	• Usage of sensors • Failure handling of the equipment	• Proactive or Reactive Decisions. • Periodic Maintenance. • Data Analytics for decision making.	• Employees training • Policies and procedures for competency enhancements • Overall organizational level of skills set.
**Lifecycle**			
• Document, Standardized Engineering, Manufacturing activities. • Approval of Processes changes.			
**Business Criticality**	**Connectivity**	**Strategic Planning**	

• Criticality of Activities in operations	• End-to-End Data collection. • Data sharing to information systems. • Main mode of connectivity • Level of integration to IT systems such as DSS, MIS, ERP and PLM.	Long term Vs Short term. Planning based on strengths or competition.	
**Failure Handling**			

• Strategy Planning & Mitigation Issues			
**Design**		

• 2D/3D Data Visualization • Digital tools (CAD/CAM)

## Practical products life-cycle of digital-twin

This section is mainly focusing on the different stages about how the digital-twin can be started from idea to end-added values services and what are the expected added-values capabilities of the digital-twin as end developed service. Also, the discussion is extended to include the different scopes and levels that the digital-twin can be utilized for.

First; digital-twin can be started as an idea or concept that can considerably enhancing the processes in an organization. This idea need to be thoroughly investigated to determine it is real feasibility for that organization therefore, feasibility study need to be conducted here to fully understand the potential benefits of the technology implementation or if the return-of-investment shows no need for such technology adoption. If the feasibility study shows a good return-of-investment, then the next phase will be the design to adopt this technology in that particular organization. The design phase includes the processes of validation, simulation capabilities and virtualization functionalities. The next phase is to put such design in production and the processes expected to be involved here are production planning, assembly as integration from multiple production lines and then the delivery of the final products. System integration is the scaling-up of the digital-twin technology to be integrated across multiple-stages. Diagnostic and troubleshooting capability to maintain the good conditions of the digital-twin functionalities should be considered and finally, the final digital-twin services as prognostics and health management (PHM) of the productions and the products [30].

The scope and the complexity of digital-twin implementation can be considered as the following aspects: It could be at the level of equipment or system level also at the level of the entire environment to be simulated. It could be at the level of system and environment or systems of systems so this how digital-twin mostly are look like as one twin or multiple twins together systems and environments or systems of systems [31].

The digital-twin added values can be described as: added monitoring functionalities. As the digital-twin is replica of the physical counterparts, the digital-twin allows to track what happening to the physical-assets in real-time and the performance of the physical-assets operations. From monitoring functionalities to predictive capabilities, the digital-twin can be utilized to mitigate operational and machine failure by sending early notification warning and this lead to reduced down-time of the systems and machines. Visualization functionality is one of the important add-value of digital-twin technology and that can be particularly beneficial as utilization of virtual reality for training purposes and how to arrange the equipment in the best physical layout. From virtual reality to augmented reality with the support of artificial intelligence/machine learning capabilities, the digital-twin can add the value of interactive functionalities by allowing the control of the machine virtually to avoid hazardous situations dealing to the machine as physically and directly also, this can be useful in term of saving and extending the life of the machines. With the power of digital-twin the manufacturing system can have sort of cognitive capabilities as well as it can be easily integrated so that allowing the assembly lines to be formed accurately and quickly. Maintenance is also a clear added-value service of the digital-twin successful adoption and implementation as the ability to check the operational efficiency of the system and to understand and also to predict information to carry-out repairs needed for the machines. Here can be said the maintenances can be planned and that's lead the system to work better as repair procedures can be timely carried-out [32].

Extending the training capabilities is one of the interesting features of implementing the digital-twin. Training through digital-twin is safe as this technology considered as the mirror of the real-life scenarios. Remote machine setup and remote troubleshooting can be performed through the digital-twins giving and excellent opportunity for safe training. Communication between all the team to ensure the products and productions issues can be done in alternatives way for optimized solutions is also a very useful and added-value service of the digital-twin implementation. At the end. It is clearly that, the digital-twin main added-value is that, it helps organizations to meet their strategies and strategic goals by reducing the operational processes hazards and costs and also allowing high-degree of flexibilities in production and products to be aligned to future demands and customers’ expectations.

## Modelling the adoption of digital-twin in manufacturing businesses

**Fig. 3:**
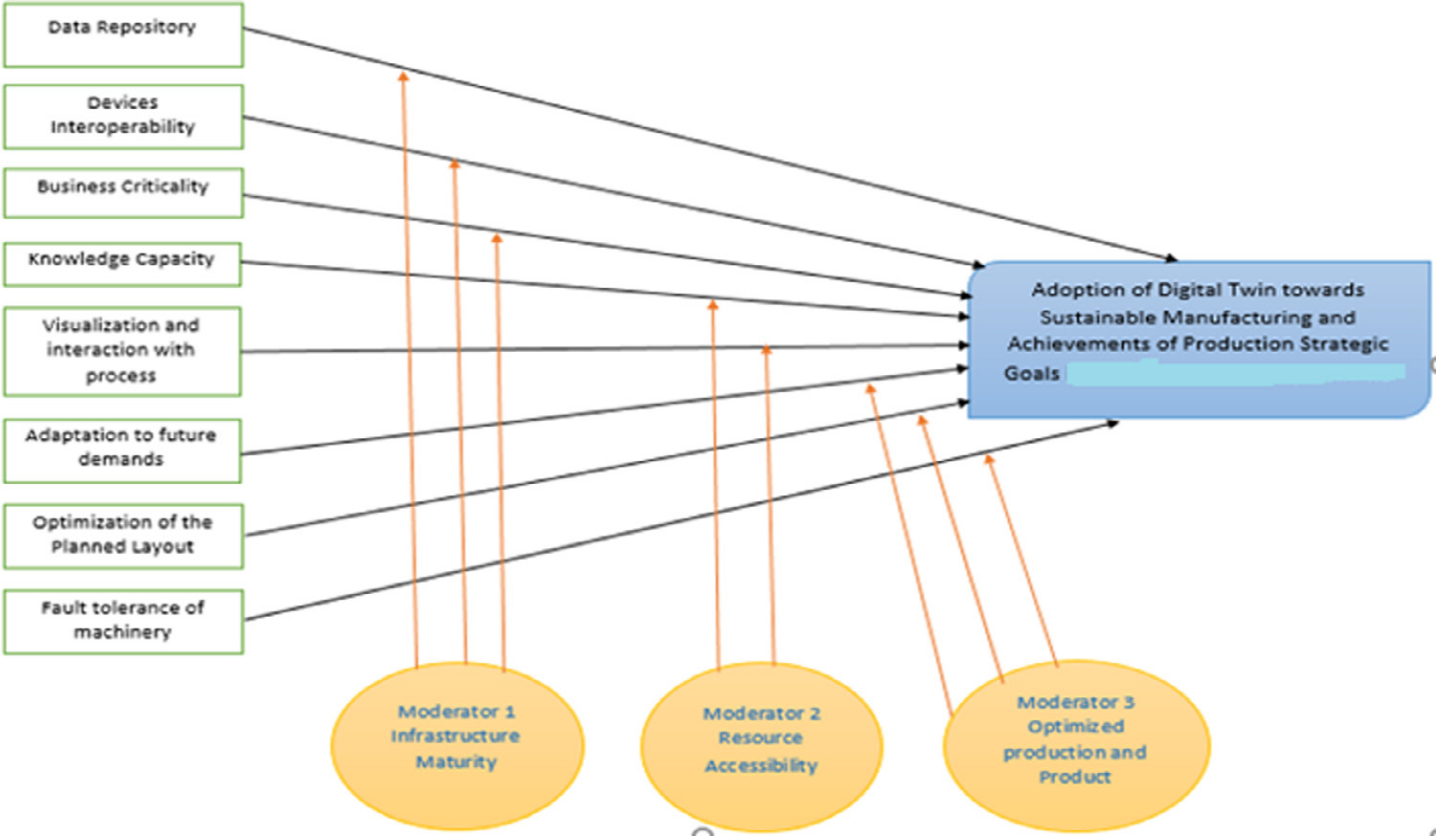
Digital-Twin Adoption for Optimized product-production.

Based on the factors discussed on previous sections to achieve successful adoption of digital-twin in manufacturing industries, a model of independent factors to achieve the targeted adoption can be visualized as Fig. 3. Moderator factors can significantly increase the direct relation between influential and the targeted factors as depicted in Fig. 3. These moderator factors are namely infrastructure maturity, resource accessibility and optimized production & products.

The specifications of the proposed model methodology of successful digital-twin adoption can be stated as the following -2 of specifications.

## Conclusions

Digital-twin technology has recently witnessed considerable interests in both of industry and academia. It is rapidly developing technology and getting more and more feasible to be implemented therefore, the successful adoption of this technology particularly in manufacturing can come with many aspects of operational efficiencies and processes optimization. It is important for manufacturing enterprises to start investigating the benefits and enhancements that they can gain by the successful adoption of the digital-twin. This paper considered as guidance to startup and to establish a solid adoption of digital-twin with feasible and high return-of-investment. It is clearly that, the adoption feasibility should be clearly understood and vision as industrial organizations are varied in size, operational complexity and strategic goals. To make the best use of this technology and to achieve high-return from implementing it, it's always necessary to understand which adoption strategy is the best to be followed from the early stage up to the implementation with full-potential. This paper aid the efforts in each manufacturing enterprise to determine first if the digital-twin technology is the good choice for them to be implemented then what is the strategy of adoption to be followed and how to reach to the full potential of this technology for optimized processes as well as in terms of being flexible for products and products to meet future markets demands as well as to achieve the strategic goals to ensure sustainability in market.

## Declaration of Conflicting Interest

The authors that they have no known competing financial interests or personal relationship that could have appeared to influence the work presented in this paper

## Data Availability

No data was used for the research described in the article.
